# Immune Characteristics and Prognosis Analysis of the Proteasome 20S Subunit Beta 9 in Lower-Grade Gliomas

**DOI:** 10.3389/fonc.2022.875131

**Published:** 2022-07-19

**Authors:** Junzhe Liu, Xinyu Yang, Qiankun Ji, Lufei Yang, Jingying Li, Xiaoyan Long, Minhua Ye, Kai Huang, Xingen Zhu

**Affiliations:** ^1^ Department of Neurosurgery, The Second Affiliated Hospital of Nanchang University, Nanchang, China; ^2^ Institute of Neuroscience, Nanchang University, Nanchang, China; ^3^ Jiangxi Key Laboratory of Neurological Tumors and Cerebrovascular Diseases, Nanchang, China; ^4^ Department of Comprehensive Intensive Care Unit, Second Affiliated Hospital of Nanchang University, Nanchang, China; ^5^ East China Institute of Digital Medical Engineering, Shangrao, China

**Keywords:** lower-grade glioma (LGG), prognostic biomarker, immunotherapy, clinical prognosis prediction, overall survival, proteasome, PSMB9

## Abstract

Glioma is a common intracranial malignancy in adults and has a high mortality due to its poor prognosis and high recurrence rate. Dysregulation of protein degradation is one of the main promoting factors in glioma development. As an indispensable unit of the proteasome, Proteasome 20S Subunit Beta 9 (PSMB9) is one of the major enzymes in ubiquitin-dependent protein degradation in cells. In addition, proteasomes also participate in a series of cellular processing, like immune regulation, nerve signal transduction, material transport through channels, cell adhesion, and various signaling pathways. However, the relationship between the PSMB9 expression and the occurrence of lower-grade glioma (LGG) is still unknown. First, we collected the RNA-seq and clinical information about LGG clinical samples from The Cancer Genome Atlas (TCGA) cohort, Chinese Glioma Genome Atlas (CGGA; including CGGAseq1 and CGGAseq2) cohort, and Gene Expression Omnibus (GEO; GSE16011, GSE61374, and Rembrandt) cohort. Then, these data were used for differential analysis, survival analysis, enrichment analysis, clinical model construction, etc. In addition, we combine immune-related data for immune-related analysis, including immune infiltration and immunotherapy. Through the above research, we have provided a new biomarker for LGG prognosis prediction and more comprehensively explained the role of PSMB9 in the development of LGG. This study determined that PSMB9 can be used as an immunotherapy target through the analysis of immune data, providing new ideas for the clinical treatment of LGG.

## Introduction

Gliomas are the major cause of central nervous system tumors that cause death in patients, which occupy half of the primary intracranial tumors and always occur in adults (1). According to the different degrees of tumor malignancy, the WHO system divides gliomas into four grades (2, 3). Because of the good prognosis of grade 1 gliomas, we regard them as benign tumors and usually do not include them when conducting glioma research. Recent evidence has shown that grades 2 and 3 gliomas have similar molecular features and fewer prognostic differences, so The Cancer Genome Atlas (TCGA) database classifies grades 2 and 3 gliomas as lower-grade gliomas (LGGs) (4); glioblastoma (GBM) has been studied alone due to its high degree of malignancy and poor prognosis (5). During the analysis, we found that there is a very large heterogeneity between LGG and GBM, so we consider that it is unreasonable to perform an integrated analysis (6, 7). Therefore, we separately conducted our study in LGG cohorts. Due to GBM’s high degree of malignancy, the common clinical treatments are surgical resection supplemented by radiotherapy and chemotherapy, but these strategies have failed to achieve the desired results (8, 9). Given the fact that clinical treatments of gliomas are not satisfactory, we have come up with novel immunotherapies that garnered positive responses, so it is urgently needed to seek new therapeutic targets and prognostic biomarkers (10, 11). As an indispensable component of the proteasome, PSMB9 is closely related to a variety of biological behaviors by participating in the protein degradation process. Our survey provided conclusive evidence for the potential of PSMB9 to act as a robust biomarker to predict the prognosis for patients in LGG.

PSMB9 is an integral member of the proteasome B-type family, involved in the composition of the proteasome. Proteasomes widely exist in the cytoplasm of eukaryotic cells and are involved in protein degradation through the non-lysosomal pathway supplemented by ATP/ubiquitin cleavage (12, 13). PSMB9 is particularly significant for the inactivation and degradation of various proteins in cells. The PSMB9 expression level in tumor tissues is much higher than in para-carcinoma tissue, suggesting that the expression of PSMB9 is highly correlated with LGG and affects various pathways in a subtle and complex way. Through subsequent analysis, we found a high correlation between PSMB9 and immunity in tumors. PSMB9 participates in a variety of biological processes and accelerates the progression of cancer. In view of its versatility, no studies have shown its mechanism of action in the pathogenesis of LGG and its potential application as a clinical immunotherapy target. By examining several databases, we have revealed its role in tumors and potential as an immunotherapy target, demonstrating a stable prognostic marker that can be used as a therapeutic target to alter the course of LGG.

Our research mainly analyzed the relationship between PSMB9 gene expression and characteristics among tumor samples through bioinformatics. We used the TCGA database as the training set and made a preliminary conclusion. Then Chinese Glioma Genome Atlas (CGGA) and Gene Expression Omnibus (GEO) databases were analyzed as the validation set to prove the universality and accuracy of the above conclusions again. We performed a correlation analysis by matching the clinical information and PSMB9 expression levels, and nomograms were constructed to predict the overall survival (OS) of patients. Through the analysis of the RNA-seq data in the low-high expression group of PSMB9, differentially expressed genes (DEGs) were screened out for functional and pathway analysis. Function annotation was conducted through Gene Ontology (GO) analysis and Kyoto Encyclopedia of Genes and Genomes (KEGG) analysis. Gene set enrichment analysis (GSEA) was performed to identify the hallmark gene sets and reveal their enrichment pathways, which can roughly predict gene functions and their roles in tumor progression (14–16). By examining the expression relationships between PSMB9 and several well-known immune checkpoints in different expression groups, we further identified a robust association between the expression of PSMB9 and the immune system. Finally, we explored the relationship between PSMB9 expression in the immunotherapy cohort and sample response in two immunotherapy databases, providing effective guidance for the application of PSMB9 as an effective prognostic marker in LGG immunotherapy. Through the above analysis, we identified that PSMB9 can be used as the prognostic marker; immune analysis was also implemented to confirm the relationship with tumor immune environment and its prospect in tumor immunotherapy application in patients with LGG.

## Materials and Methods

### Acquisition and Preprocessing of Lower-Grade Glioma Datasets

The LGG data were mainly downloaded from three public datasets for the analysis of the whole article, including TCGA, CGGA, and GEO databases. The mRNA sequencing and clinical information of the LGG samples were downloaded from the Genomic Data Commons Data Portal website (https://portal.gdc.cancer.gov/). Analogously, the data of the CGGA and GEO databases can be downloaded from the CGGA website (http://www.cgga.org.cn/) and GEO website (https://www.ncbi.nlm.nih.gov/gds/). In the GEO dataset, GSE16011, GSE61374, and Rembrandt cohorts were selected for this research. After the original data were obtained, the samples were screened according to the predesigned inclusion criteria to pick the meaningful samples. The specific inclusion criteria are as follows: 1) patients with primary glioma were chosen for the analysis; 2) grade 2 and 3 glioma patients according to the WHO classification; 3) the mRNA expression of each gene in this sample was clear without deletion value; 4) the minimum follow-up time of patients was more than 30 days, and there were no missing values of basic information such as age and sex; and 5) there is no missing value of each gene information, such as 1p19q co-deletion, IDH status (17), MGMT promoter methylation (18). The summary of the proportion of clinical features is shown in **Table 1**. A total of 473 LGG samples out of 529 remained after TCGA dataset was filtered. The original RNA-seq data were fragments per kilobase of transcript per million fragments mapped (FPKM) data, and a calculation formula that was previously publicly available was used to convert them into transcripts per kilobase million (TPM) data for bioinformatics analysis to make the obtained results more convincing (19–21). In addition, the RNA-seq data and clinical information of IMvigor210 (n = 398) and Gide2019 (n = 41) were downloaded from the GEO dataset to verify the prognostic role of PSMB9 in immunotherapy (22, 23).

**Table 1:** Summary of clinical information of LGG patients included in this study.

Characteristics	TCGA dataset (n = 473)		Meta-CGGA dataset (n = 413)		GEO dataset (n = 379)	
Age						
≥40	254	53.70%	218	52.78%	313	82.59%
<40	219	46.30%	195	47.22%	65	17.15%
PSMB9						
High	131	27.70%	91	22.03%	111	29.29%
Low	342	72.30%	322	77.97%	268	70.71%
Grade						
WHO II	229	48.41%	183	44.31%	159	41.95%
WHO Ill	244	51.59%	230	55.69%	220	58.05%
Gender						
Male	258	54.55%	238	57.63%	245	64.64%
Female	215	45.45%	175	42.37%	134	35.36%
IDH						
Wild type	85	17.97%	102	24.70%	58	15.30%
Mutant	388	82.03%	311	75.30%	159	41.95%
N/A	N/A	N/A	N/A	N/A	162	42.74%
1p19q						
Coded	156	32.98%	126	30.51%	74	19.53%
Non-coded	317	67.02%	287	69.49%	139	36.68%
N/A	N/A	N/A	N/A	N/A	166	43.80%
MGMT						
Unmethylated	82	17.34%	169	40.92%	N/A	N/A
Methylated	391	82.66%	244	59.08%	N/A	N/A

LGG, lower-grade glioma; TCGA, The Cancer Genome Atlas; CGGA, Chinese Glioma Genome Atlas; GEO, Gene Expression Omnibus.

### Potential of PSMB9 as a Prognostic Biomarker

TCGA, CGGA, and GEO cohorts were divided into two subgroups artificially. The optimal cutoff value was calculated using the R package “survival” and “survminer” in the cohort, and the subsequent studies used this criterion to group the low and high PSMB9 expression groups. The survplots were drawn from the data in three databases. Furthermore, the area under the curve (AUC) was performed to identify the prognostic accuracy of PSMB9 in three cohorts. Multivariate Cox regression analyses were also conducted to ascertain the independent prognostic ability.

### Enrichment Analysis of PSMB9

The RNA-seq data of TCGA cohort were obtained, the samples were grouped into two according to the previous grouping criteria, and 1,288 DEGs were screened out. GO and KEGG enrichment analyses were carried out through the R package “clusterProfiler” to obtain the cell functions and pathways in which DEG participated (24). At last, the tumor hallmarks enriched in LGG were sorted out by the GSEA software, and pathways with higher enrichment scores (ESs) were singled out for multi-GSEA.

### Prediction Model Construction and Accuracy Verification

Univariate and multivariate Cox regression analyses were conducted to determine the independent prognostic effect of PSMB9. Relevant clinical information was included, such as age, gender, WHO grade, IDH mutation, 1p19q co-deletion, and MGMT promoter mutation. The nomogram model was constructed based on the multivariate Cox regression analysis results using the R package “rms.” TCGA calibration curves were constructed by the “calibrate” function in the “rms” packages, and the results above were verified in the CGGA and GEO cohorts. Decision curve analysis (DCA) curves were built to better illustrate the clinical utility of the nomogram model by comparing the benefits of different variables in the model.

### Analysis of Immune Cell Infiltration in Lower-Grade Glioma

Infiltrating cell types in glioma samples were analyzed through single-sample GSEA (ssGSEA), CIBERSORT, and ESTIMATE methods and obtained the scores of corresponding immune signatures. The rank of gene expression values was standardized in a single sample, and the ES was calculated using an empirical cumulative distribution function, which is called ssGSEA (25). The r package “GSVA” was applied to evaluate the microenvironment of immune cell infiltration in LGG tumor samples (26). The median PSMB9 expression level was used as the grouping basis to divide LGG samples into high and low expression groups for ssGSEA. Tumor purity, estimated score, immune score, and interstitial score of LGG samples can be obtained through the calculation of the ESTIMATE package. In addition, the relative abundance of each type of immune cell in tumors can be obtained by ssGSEA calculation. CIBERSORT online tool (http://cibersort.stanford.edu/) was used to analyze the proportion of 22 human hematopoietic cell types cells in TCGA cohort (27). The difference analysis of the immune cell types in the PSMB9 high and low expression groups was carried out through the “vioplot” packages. In addition, a single-cell analysis of PSMB9 was also performed in the existing single-cell cohort on the Tumor Immune Single-cell Hub website (TISCH: http://tisch.comp-genomics.org/).

### Prediction of Tumor Immunotherapy

Immunotherapy is the latest progress in tumor therapy, among which antagonistic antibodies are one of the important therapeutic methods, like cytotoxic T-cell lymphocyte antigen 4 (CTLA-4) and programmed death 1 (PD-1) (28). In the IMvigor210 cohort (29), patients who received atezolizumab, an anti-PD-L1 drug, were divided into four groups based on their treatment response: complete response (CR), partial response (PR), stable disease (SD), and progressive disease (PD). By analyzing the gene expression in the cohort and its influence on the immunotherapy effect, the difference in PSMB9 expression level in LGG patients against the PD-L1 immunotherapy effect can be predicted. To further access the potential of PSMB9 as an immunotherapy target, PSMB9 expression and acceptance were also validated in another cohort (Gide2019) that was treated with nivolumab, pembrolizumab, and ipilimumab.

### Acquisition of Clinical Samples

Twelve clinical samples were collected from inpatients in the Nanchang University Second Affiliated Hospital to conduct our survey, including six clinical normal brain tissues (NBTs), three grade 2 samples, and three grade 3 samples according to WHO classification. All of the samples were stored at a temperature of −196°C immediately after surgical excision. The study has been approved by the Medical Ethics Committee of the Second Affiliated Hospital of Nanchang University. The collection and utilization of clinical samples are strictly in compliance with the established guidelines, and informed consent of patients was obtained.

### Cell Culture

SW-1088 and SW-1783 LGG cell lines used in this study were purchased from American Type Culture Collection (ATCC; Manassas, VA, USA). SW-1088 and SW-1783 were cultured with Leibovitz’s L-15 Medium (Gibco, Grand Island, NY, USA) mixed with 10% fetal bovine serum (FBS; Gibco) and 1% Pen Strep (Gibco) and at a temperature of 37°C under normoxic conditions. All the cell samples were obtained during the logarithmic growth period to ensure that the cell morphology and cell function are in the best condition for observation and processing.

### Immunofluorescence Protein Localization

To determine the functional pathway of PSMB9, the intracellular localization of PSMB9 was detected by immunofluorescence assay. SW1088 and SW1783 cells were planted on slides, immobilized with 4% paraformaldehyde for 60 min until the cells grew into a suitable density, washed with PBS several times to remove the residual paraformaldehyde, and then incubated in 0.3% Triton X-100 (dissolve in PBS) for 15 min. The cells were washed with PBS another three times, and the antigen was blocked with 5% goat serum for 1 h. Then a rabbit anti-PSMB9 antibody (14544-1-AP; 1:50; proteintech, Wuhan, China) was used to especially bind the PSMB9 protein in the cells at 4°C for no less than 12 h. Alexa Fluor 488-conjugated AffiniPure Goat Anti-Rabbit IgG was used as the secondary antibody to conjugate with the primary antibody after the primary antibody incubation is complete. Next, the sample needs to be incubated with 10 μg/ml of DAPI for 20 s in the dark. Finally, the sample was washed three times with PBS, and anti-quench sealing tablets were used for anti-quench treatment (Fluorescent Mounting Media; Bioss, Woburn, M, USA; Cat. C02-04003) if necessary, followed by confocal microscopy (Leica, Wetzlar, Germany).

### Western Blotting of Clinical Samples

The tissue samples were ground and crushed after being cleaned with PBS and then digested and lysed with radioimmunoprecipitation assay (RIPA) lysate dissolved with 1% phenylmethylsulfonyl fluoride (PMSF) on ice for 30 min. After the samples were completely lysed, they were centrifuged in a high-speed refrigerated centrifuge at 12,000 rpm for 15 min, and the tissue protein was obtained. The concentration of the tissue protein was measured with the bicinchoninic acid (BCA) kit (Beyotime, Shanghai, China). Then 10% sodium dodecyl sulfate–polyacrylamide gel electrophoresis (SDS-PAGE) was applied to separate the protein lysates, and 20 ng of total proteins was added to each lane. The protein in the SDS-PAGE glue is transferred to the polyvinylidene difluoride (PVDF) membranes by membrane transfer operation. A 5% bovine serum albumin (BSA) solution was used to block the antigen epitope on the membrane for 2 h at room temperature. Then, the membrane was treated with the primary antibody (PSMB9, 1:2,000, proteintech, Cat. 14544-1-AP; Tubulin, 1:5,000, proteintech Cat. 11224-1-AP) and secondary antibody (horseradish peroxidase (HRP)-conjugated anti-rabbit secondary antibody, 1:5,000, proteintech, Cat. 15001). Enhanced chemiluminescence (ECL; UE; Cat. S6009M) kit was used to visualize the position and abundance of PSMB9 protein. Tanon-5200 Multi was used to visualize the protein brand. The final image was read with ImageJ software (version 1.52a) for gray value, and Tubulin was used as an internal reference control for semi-quantitative statistical analysis of the expression of the target protein.

### Fluorescence Immunohistochemistry of Clinical Samples

Previous studies have shown that there is a significant relationship between PSMB9 protein and CD8+ T cells. In order to further detect the relationship between PSMB9 and CD8+ T cell infiltration in tumor tissues, fluorescence immunohistochemistry (FIHC) was performed on tumor samples. The effect of PSMB9 on the infiltration of CD8+ T cells in tumor tissues was revealed by detecting the CD8 and PSMB9 proteins. Five tumor tissue samples were obtained from the biological sample bank of the Second Affiliated Hospital of Nanchang University. First, the tumor tissue was embedded in paraffin, and the sample was cut into 4–6 μm thick slices with a microtome. The samples were placed on glass slides and incubated in xylene solution for 15 min twice, 100% ethanol for 10 min, 85% and 75% ethanol for 5 min for deparaffinization, and antigen was recovered in EDTA buffer (PH 8.0). A 3% BSA solution was used to incubate the slices for 0.5 h to block the antigen, and then anti-CD8 (1:100) and anti PSMB9 (1:100) antibodies were used as the primary antibody to incubate the slices overnight. The residual primary antibody was cleaned with PBS solution, and Cy3 (1:300) and AF 488 (1:400) were used as secondary antibodies for fluorescent labeling and incubated in the dark for 1 h. Finally, the nuclei were stained using DAPI dye for localization. The slides were observed and photographed with a fluorescence microscope.

### Statistical Analysis

**Figure 1:**
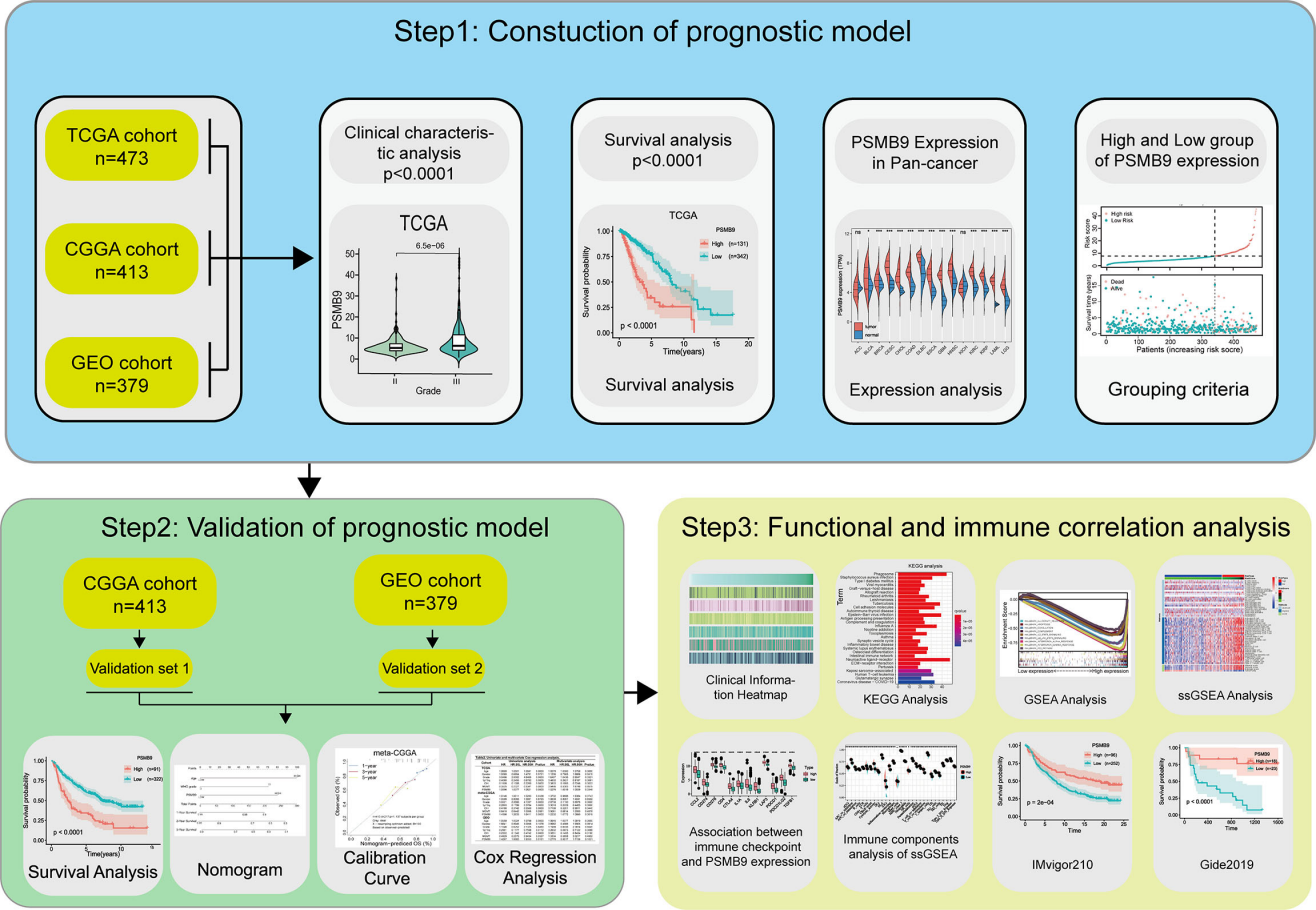
Flow chart of this study. Step1: The sequencing data and clinical information data of LGG patients were downloaded from the TCGA, CGGA and GEO databases respectively, and the PSMB9 gene expression was extracted and matched with the clinical information for analysis of survival and expression differences. Step2: Nomogram model was constructed and its predictive stability was tested for the prognose in LGG patients. Step3: GO, KEGG, and GSEA analysis were performed to predict the function of PSMB9. Immune-related analyses including ssGSEA, CIBERSORT and ESTIMATE were performed to verify the correlation of PSMB9 with the immune environment and its potential in immunotherapy was identified in two independent immunotherapy cohorts(IMvigor210 and Gide2019).

An OS analysis was conducted by the Kaplan–Meier method (two-sided log-rank test) to analyze the clinical characteristics of two LGG subgroups with high and low PSMB9 expressions. The optical cutoff value was sorted out so that the samples can be divided into two subgroups at that point, and the p-value was the lowest when the “survminer” package was used (30). The optical cutoff value was used as the basis for grouping the dataset into two subgroups in the following study. The predictive potential of PSMB9 mRNA can be accessed by time-dependent receiver operating characteristic (ROC) curves, and the ability was compared by calculating the AUC values. A Students’ t-test was performed to calculate the value of immune-related factors including immune score, stromal score, and tumor mutational burden (TMB) between the low and high PSMB9 expression subgroups, which were artificially divided. Moreover, univariate and multivariate Cox regression analyses were performed through the R package “rms” to evaluate the effectiveness of PSMB9 as an independent prognostic factor, and the nomogram model was constructed based on the above results. The C-index and calibration plot were used to consolidate the above results. All the statistics and methods mentioned above were processed with R software (version 4.0.4, http://www.r-project.org/), Perl (version 5.24.3), and SPSS 22.0 for windows (SPSS, Chicago, IL, USA). The flow chart of this study for performing the data analysis is shown in **Figure 1**.

## Result

### The Difference in PSMB9 Expression in Tumor Tissues

**Figure 2:**
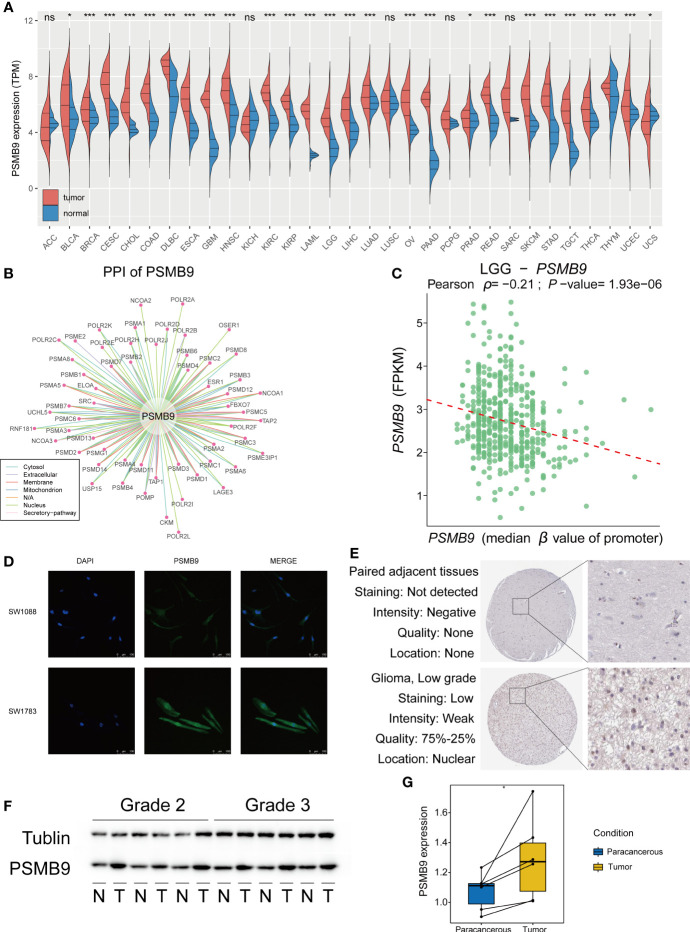
Overview of PSMB9 protein in tumor samples. Basic expression, characteristics and distribution of PSMB9. **(A)** PSMB9 protein is differentially expressed in most tumors and is usually highly expressed in tumor tissues. **(B)** The PPI network of PSMB9. (Data derived from Pubmed). **(C)** The relationship between the level of promoter methylation and PSMB9 expression shows a higher PSMB9 expression correlated with a lower promoter methylation level. **(D)** Confocal images verify the distribution of PSMB9 in SW-1088 and SW-1783 cells and indicate that PSMB9 protein was expressed in both nucleus and cytoplasm. **(E)** The difference of PSMB9 protein expression and location between normal and tumor tissues were detected by immunohistochemistry which suggested that the expression level of PSMB9 was significantly increased in tumor tissues (Images were downloaded from Human Protein Altas). **(F)** WB verifies PSMB9 protein expression differences in clinical tissue samples. Tumor tissues have a higher PSMB9 expression than normal tissue. **(G)** ImageJ detects protein gray values for paired tests to determine protein expression differences.

We explored the expression of PSMB9 in pan-cancer using TCGA and GTEx datasets, and the results show that PSMB9 was upregulated in most tumors, including LGG (**Figure 2A**). We performed the protein–protein interaction (PPI) network through the data from the comPPI website (http://comppi.linkgroup.hu/), and each kind of PPI can be traced back to PubMed. The results show that there are many kinds of proteins that interact with PSMB9 at different subcellular locations in the cell, such as the cytosol extracellular, membrane, and nucleus (**Figure 2B**). We downloaded the gene expression and DNA methylation data of TCGA database from UCSC Xena (http://xena.ucsc.edu), and the “DESeq2” package was employed to normalize and visually analyze the raw data. We realized that the higher the methylation level of the PSMB9 promoter, the harder it is for transcription factors to bind the PSMB9 promoter region, and the expression will decrease (**Figure 2C**). To observe the abundance and distribution of PSMB9 protein in LGG cells, we performed the immunofluorescence assays in SW-1088 and SW-1783 LGG cell lines. We found that the protein is distributed in the nucleus and cytoplasm, which can preliminarily prove that the protein can play a role in both the nucleus and cytoplasm (**Figure 2D**). In order to further verify the differential expression of PSMB9 protein in normal and tumor tissues, we downloaded immunohistochemical pictures of LGG samples from the Human Protein Atlas (HPA; https://www.proteinatlas.org/). The results show that the expression of PSMB9 is much more than that in NBTs (**Figure 2E**). Finally, we performed Western blotting (WB) with 12 clinical samples to identify the expression of PSMB9. It was proven that the higher the WHO grade, the higher the protein expression of PSMB9 (**Figure 2F**), which is in accordance with the conclusions we have drawn from the statistical analysis of the database. According to the WB results, we used ImageJ to calculate the corresponding gray value to represent the expression level (**Figure 2G**). All the evidence mentioned above proves that the expression of PSMB9 in tumor tissues is higher than that in normal tissues.

### Clinical Features of PSMB9 Expression

**Figure 3:**
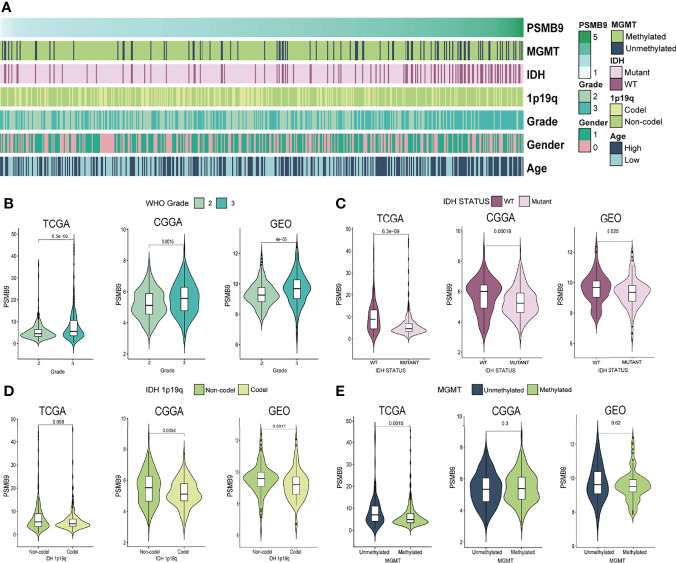
PSMB9 correlated with the clinical and molecular characteristics of gliomas. **(A)** Heatmap reveals the relationship between various clinical features in each sample of different PSMB9 expressions. **(B–E)** The violin plot shows the significant difference in WHO Grade **(B)**, IDH status **(C)**, IDH 1p19q **(D)** and MGMT **(E)** in different PSMB9 expression subgroups.

We downloaded the clinical characteristic data of each sample from TCGA database and analyzed their differences through the grouping obtained previously. We sorted the samples according to the expression level of PSMB9; however, some values are so high that it is difficult to distinguish the expression differences in other samples with smaller expression levels. Therefore, we use log_2_(TPM + 1) transformation to logarithmically process them to reduce the disparity in expression levels between samples. As for clinical information, we made a heatmap of the correlation between expression level and clinical features (**Figure 3A**). We can observe the different expressions between the status of clinical features and judge if there is a significant statistical difference based on the p-value. The results show that there are statistical differences in the expression of PSMB9 in different WHO classifications and IDH statuses. However, there is no clear difference between the 1p19q co-deletion in TCGA database and the methylation of the MGMT promoter in the GEO database (**Figures 3B–E**).

### Survival Analysis Based on the PSMB9 Expression

**Figure 4:**
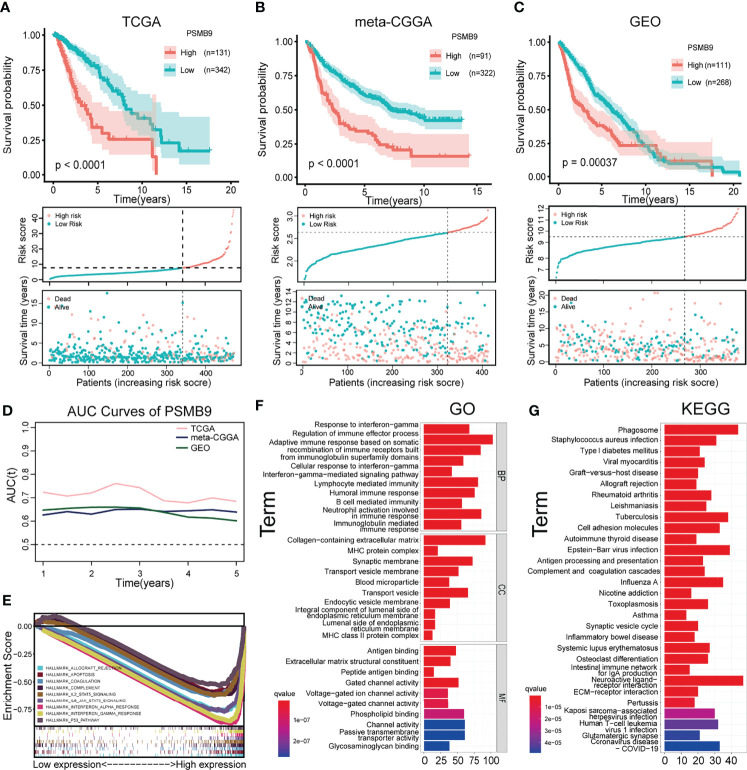
Survival analysis and enrichment analysis. **(A–C)** K-M survival analysis was performed to validate that shorter survival and poorer prognosis in LGG patients with high PSMB9 expression in three databases (TCGA **(A)**, CGGA **(B)** and GEO **(C)**). **(D)** AUC curve values were all above 0.6, indicating that the predictive power of PSMB9 was stable **(E)** Multi-enrichment analysis of PSMB9 related DEGs. **(F)** Gene Ontology analysis of PSMB9-related DEGs. **(G)** Kyoto Encyclopedia of Genes and Genomes pathway analysis of PSMB9 related DEGs.

After screening according to the aforementioned screening criteria, we retained 473, 413, and 379 samples in TCGA, CGGA, and GEO databases, respectively. By analyzing the mRNA-seq data of TCGA dataset, the expression level of PSMB9 gene was extracted and analyzed as well as the survival time and survival state of patients. After statistical analysis, we sorted the optimal cutoff value and drew the relevant survplot. The figures indicated that among the groups of different PSMB9 expression levels, there were significant differences in patient survival, and samples with high PSMB9 expression tended to have poor prognoses (**Figure 4A**). The same conclusion is also confirmed in the validation set of the CGGA and GEO databases (**Figures 4B, C**). However, the situation is not the same in other tumors. For example, the patients with higher PSMB9 expression tend to have better prognoses in BCLA, BRCA, OV, SARC, SKCM, and THCA (**Supplementary Figures 1**, **2**), which is contrary to the conclusion in LGG. This indicates that different types of tumors contain different tumor environments, resulting in different functions of PSMB9 involved. In addition, we verify the accuracy of the PSMB9 prognostic model in the three datasets of TCGA, CGGA, and GEO. The results show that it is robust and convincing in the analysis of 1-, 3-, and 5-year survival rates. The AUC values were totally beyond 0.6, which proves the accuracy of the clinical prognosis model (**Figure 4D**). The above prognostic-related analysis results indicate the feasibility of PSMB9 as a prognostic-related marker in LGG patients.

### Differentially Expressed Gene Enrichment Analysis of PSMB9

We obtained the DEGs between the high and low PSMB9 groups through the website Morpheus (https://software.broadinstitute.org/morpheus/), and the enrichment analysis was conducted based on the above results. GO analysis proved that the close connection between DEGs and immune-related biological behavior and the DEGs of PSMB9 were enriched in the regulation of the immune effector process and neutrophil activation involved in immune response (**Figure 4F**). KEGG analysis identified that DEGs of PSMB9 were highly related to the phagosome and neuroactive ligand–receptor interaction and many other enrichment analysis results (**Figure 4G**). The above enrichment analysis results prove the function of PSMB9 in the nervous system and its connection with immunity, which further verifies its role in the occurrence of brain tumors and also reveals that there must be some relationship between PSMB9 and tumor immunity. These inevitable connections provide guidance for our subsequent immune-related analysis of PSMB9. GSEA was performed to reveal detailed information on enrichment results such as “interferon gamma response” and “IL6 JAK STAT3 signaling” in the whole LGG samples in TCGA dataset. A multi-GSEA was conducted to compare the DEGs in different pathways clearly (**Figure 4E**).

### Construction of Nomogram Model and Clinical Prediction Model

**Figure 5:**
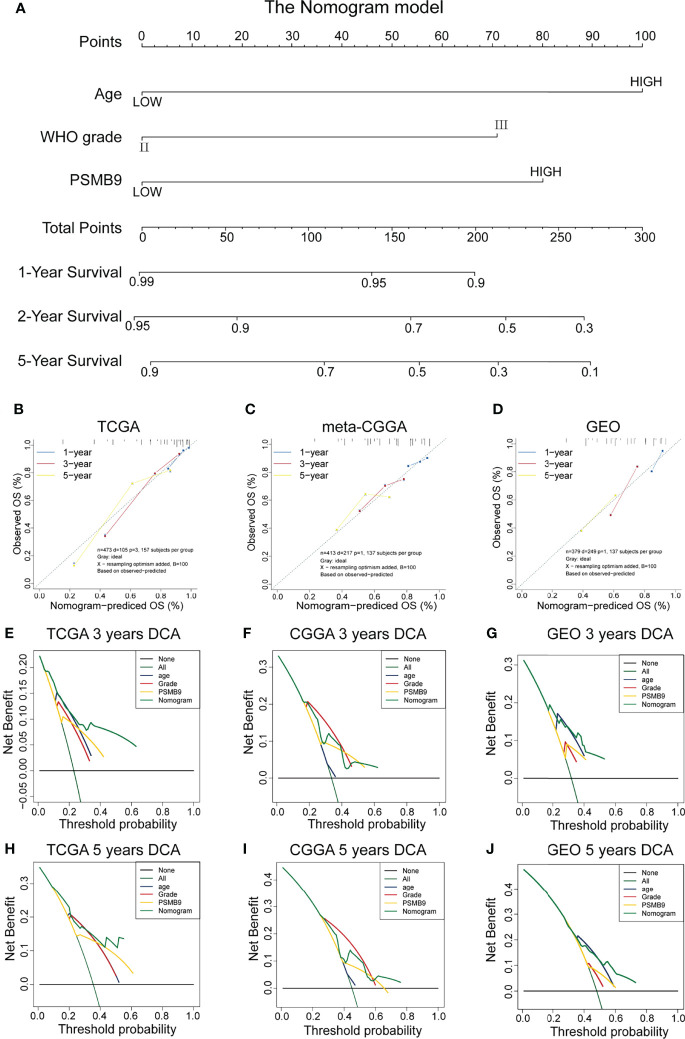
Construction and validation of the nomogram model. **(A)** Construction of Nomogram Model about clinical features (including WHO Grade, IDH Status, IDH 1p19q and MGMT) in patients with low-grade glioma. **(B–D)** The 1/3/5-year OS calibration curves verify the prediction accuracy of the PSMB9-based nomogram model in the TCGA(B), meta-CGGA **(C)** and GEO **(D)** LGG cohorts. **(E–J)** Decision Curves Analysis (DCA) construction in TCGA **(E, H)**, meta-CGGA **(F, I)** and GEO **(G, J)**.

We collected the mRNA sequencing data of LGG patients downloaded from TCGA dataset and performed screening according to the aforementioned criteria. TCGA dataset (n = 473) was regarded as the training dataset, and the CGGA (n = 413) and GEO (n = 379) datasets were used as the validation dataset. We conducted univariate and multivariate Cox regression analyses to verify the robustness of the PSMB9 expression as an independent prognostic biomarker (**Table 2**). The results show that higher age, PSMB9 expression, and WHO grade were risk factors and that IDH status, 1p19q co-deletion, and MGMT were protective factors in the three LGG cohorts. Furthermore, the nomogram model was constructed to evaluate the clinical application of PSMB9 as a prognostic biomarker with three risk factors (age, WHO grade, and PSMB9 expression level) confirmed in the multivariate model (**Figure 5A**). Moreover, we identified the accuracy of the nomogram by calculating the C-index of each cohort (TCGA:0.762; CGGA:0.678; GEO:0.671), and calibration curves indicated that the nomogram based on PSMB9 expression has a robust prediction ability in 1/3/5-year prognosis (**Figures 5B–D**). The DCA curves showed that in several datasets, the predictive ability of the nomogram model for 3/5-year survival was significantly higher than that of other prognostic factors. In addition, at a higher threshold, the prediction ability of the nomogram model is better than that of other factors (**Figures 5E–J**).

**Table 2:** Univariate and multivariate Cox regression analyses.

Cohort	Univariate analysis				Multivariate analysis			
	HR	HR.95L	HR.95H	p-Value	HR	HR.95L	HR.95H	p-Value
**TCGA**								
Age	1.0669	1.0501	1.0841	0.0000	1.0578	1.0390	1.0768	0.0000
Gender	1.0069	0.6854	1.4791	0.9721	1.1295	0.7565	1.6866	0.5515
Grade	3.0486	2.0002	4.6465	0.0000	1.6487	1.0436	2.6047	0.0321
1p/19q	0.4080	0.2455	0.6782	0.0005	0.4632	0.2621	0.8187	0.0081
IDH	0.1509	0.1008	0.2260	0.0000	0.3803	0.1995	0.7248	0.0033
MGMT	0.3525	0.2327	0.5341	0.0000	0.9495	0.5383	1.6748	0.8579
PSMB9	1.0596	1.0377	1.0821	0.0000	1.0276	1.0019	1.0539	0.0348
**Meta-CGGA**								
Age	1.0146	1.0011	1.0283	0.0338	1.3702	0.9696	1.9364	0.0743
Gender	1.0599	0.8093	1.3881	0.6725	1.0659	0.8091	1.4041	0.6502
Grade	3.0521	2.2666	4.1097	0.0000	2.8738	2.1190	3.8976	0.0000
1p/19q	0.2603	0.1799	0.3764	0.0000	0.3018	0.2039	0.4465	0.0000
IDH	0.4180	0.3142	0.5560	0.0000	0.7180	0.5201	0.9911	0.0440
MGMT	0.8416	0.6442	1.0995	0.2061	0.9061	0.6814	1.2050	0.4978
PSMB9	1.4398	1.2633	1.6411	0.0000	1.2232	1.0772	1.3890	0.0019
**GEO**								
Age	1.0559	1.0324	1.0799	0.0000	1.0645	1.0377	1.0919	0.0000
Gender	1.6641	0.8945	3.0956	0.1078	0.9962	0.4986	1.9905	0.9914
Grade	1.1546	0.6252	2.1325	0.6460	1.1989	0.6036	2.3814	0.6045
1p/19q	0.2991	0.1177	0.7598	0.0112	0.2632	0.0973	0.7122	0.0086
IDH	0.2522	0.1342	0.4742	0.0000	0.3551	0.1495	0.8434	0.0190
MGMT	0.4929	0.2575	0.9434	0.0327	1.3304	0.5858	3.0217	0.4952
PSMB9	1.4267	1.0683	1.9053	0.0161	1.2770	0.9517	1.7136	0.1031

TCGA, The Cancer Genome Atlas; CGGA, Chinese Glioma Genome Atlas; GEO, Gene Expression Omnibus.

### Relationship Between PSMB9 and Immune Checkpoint Expression

**Figure 6:**
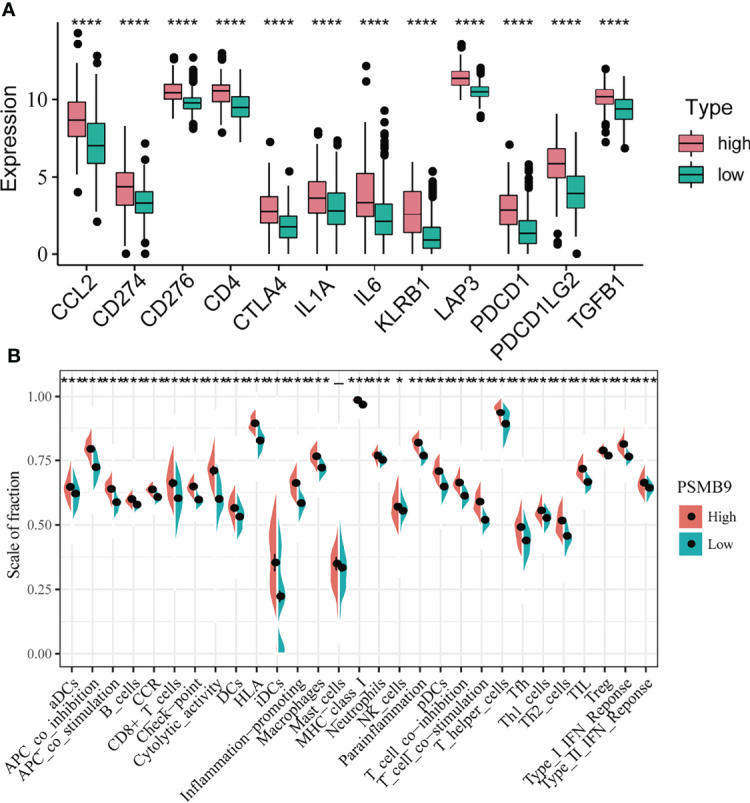
Differential analysis of immune characteristics between high and low PSMB9 expression groups. **(A)** In the high-PSMB9 expression group, the expression of 12 common immune checkpoints is up-regulated, and there was significant statistical significance in the high and low expression groups of PSMB9. **(B)** In the high-PSMB9 expression group, the infiltration scale of immune cells and abundance of immune-related molecules based on the results of ssGSEA were significantly up-regulated. (ns P>0.05; *P<0.05; **P<0.01; ***P<0.001; ****P<0.0001).

KEGG and GO enrichment revealed the robust linkage between the expression of PSMB9 and the immune microenvironment, especially in the B cell-mediated immunity and interferon-gamma-associated pathways. To further our research, we shifted the focus of our research to the analysis of immune infiltration. The first thing we did is identify the relationship between the expression of immune checkpoints and PSMB9, and we extracted the mRNA-seq data of different immune checkpoints and conducted the differential analysis in the high and low PSMB9 expression subgroup of LGG samples in TCGA dataset. We concluded that the expression of 12 kinds of the immune checkpoint is usually elevated in the high expression group compared with the low expression subgroup, and all the results were statistically different (p < 0.05) (**Figure 6A**). These results suggest that PSMB9 may be involved in the regulation of immune checkpoint expression, which may promote the effective response of immunotherapy drugs to tumor cells by increasing its expression and thus improving the efficacy. However, there is still insufficient evidence to show the exact relationship between PSMB9 and immune checkpoint expression, and the regulation mechanism of PSMB9 on immune checkpoint expression needs to be further explored.

### Analysis of Tumor Immune Infiltration

**Figure 7:**
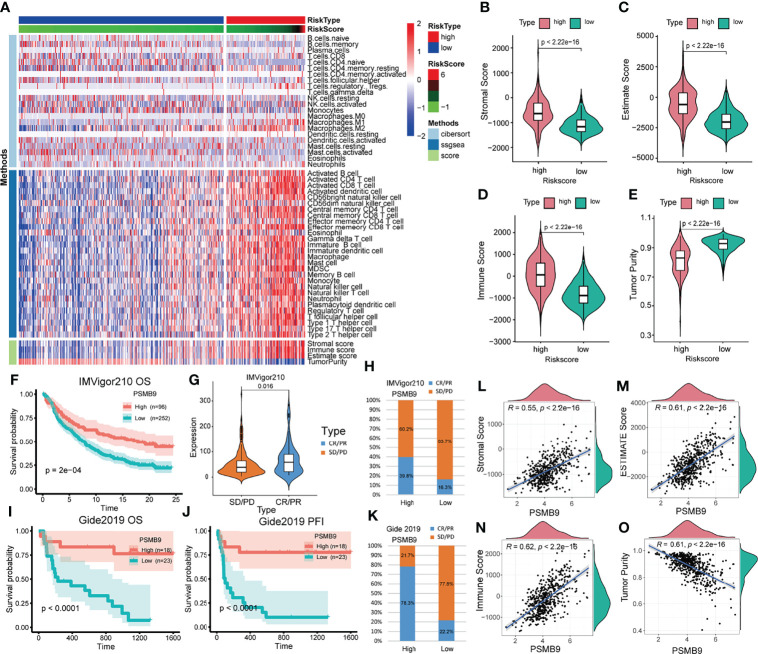
Analysis of immune-related differences and treatment prediction. **(A)** Heatmap revealed the immune infiltration in the LGG cohort through CIBERSORT, ssGSEA and ESTIMATE algorithm. **(B–E)** The relevance between PSMB9 expression and immune-related score (including Stromal score, Estimate score, Immune score and Tumor purity). **(F)** Kaplan–Meier overall survival (OS) analysis in anti-PD-L1 (IMvigor210) cohorts in PSMB9 expression high and low group. **(G)** Expression of PSMB9 in different treatment response groups. **(H)** The proportion of the difference in response to anti-PD-L1 immunotherapy in PSMB9 high and low expression group. **(I)** Kaplan–Meier overall survival (OS) analysis in Gide2019. **(J)** Kaplan–Meier progression-free interval (PFI) analysis in Gide2019 cohort. **(K)** The proportion of the difference in response to anti-CTLA4 and anti-PD1 immunotherapy in PSMB9 high and low expression group. (L-O) The scatter plot confirmed the relationship between PSMB9 expression and immune-related score.

**Figure 8:**
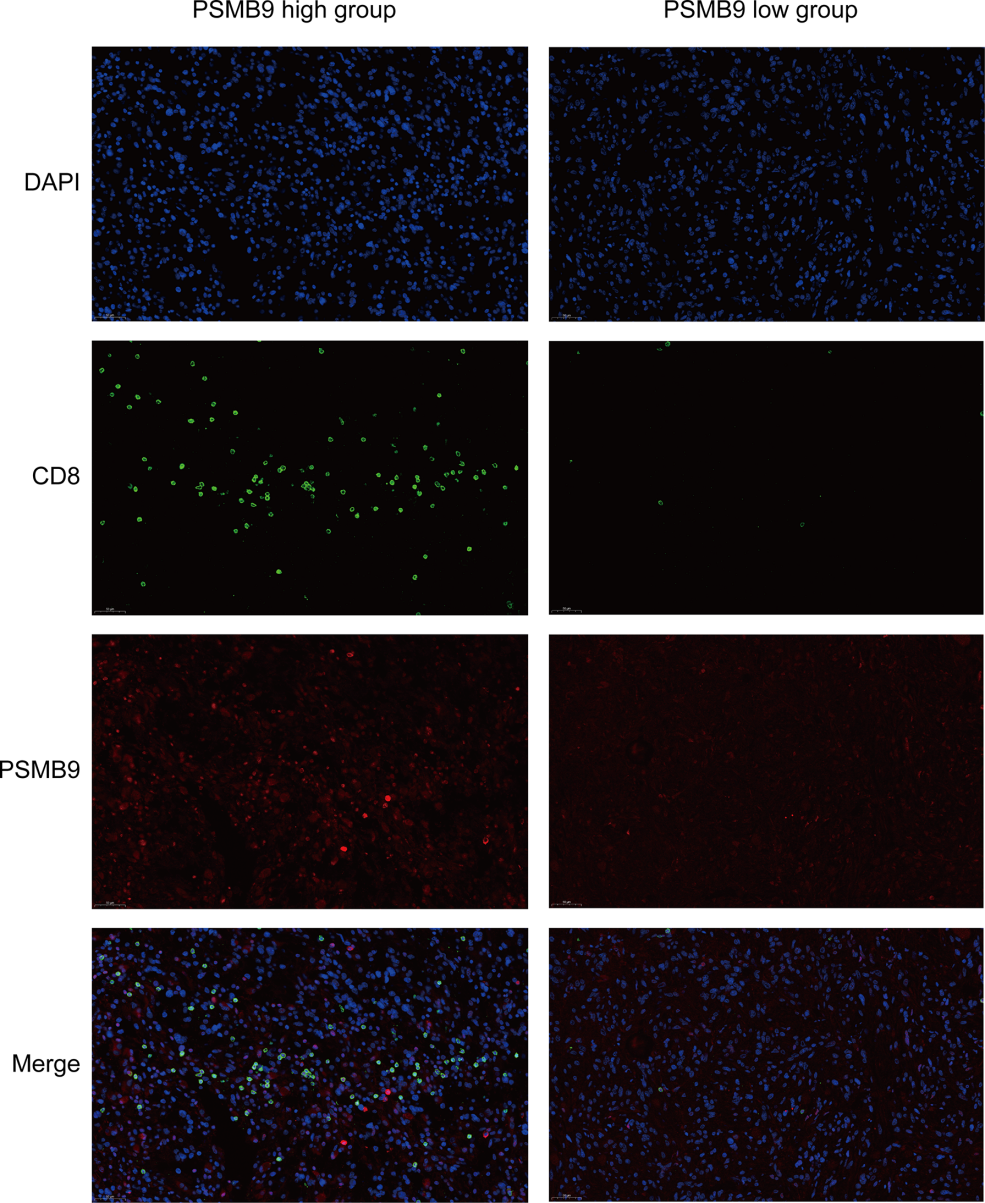
Fluorescent Immunohistochemistry of LGG tumor tissue. DAPI is used to display the localization of nucleus, green represents CD8 protein, red represents PSMB9 protein, and Merge is the overlapping of three pictures.

Since we identified the fact that the expression of immune checkpoints in the PSMB9-high group is higher than that in the PSMB9-low group, we made a further study to show the connection between PSMB9 expression and other immune items. To understand the infiltration of various subtypes of immune cells and molecules in tumors, we calculated the enrichment degree of 29 immune-related molecules and processes by the ssGSEA algorithm and conducted a correlation analysis by separating them into two subgroups according to the previous expression criteria (**Figure 6B**). We found that all immune-related signature indexes were upregulated in the PSMB9 high expression group, and the immune-related scores of most items were significantly increased in the high expression group (p < 0.05). The above results indicate that PSMB9 expression is significantly correlated with most tumor immune characteristics and also demonstrates the great potential and role of PSMB9 in the tumor immune system. Apart from ssGSEA, CIBERSORT and ESTIMATE algorithms were applied to acquire other hallmarks of immunity and tumor, like stromal score and tumor purity (**Figure 7A**). TISCH website analysis showed that PSMB9 showed high infiltration in CD8+ T cells in the glioma single-cell cohort (**Supplementary Figure 3**), indicating that PSMB9 may be helpful for CD8+ T-cell activation and chemotaxis. Then, we conducted a difference test and linear regression analysis and found that there were significant differences in each indicator between the high and low expression groups, indicating that PSMB9 plays a role in tumor immunity and has stable prognostic ability in LGG patients (**Figures 7B–E, K–N**). FIHC revealed the relationship between PSMB9 expression and CD8+ T-cell infiltration in LGG (**Figure 8**). The results showed that PSMB9 was significantly expressed in LGG tissues, and CD8+ T cells infiltrated LGG tissues with high PSMB9 expression. This is consistent with our bioinformatics conclusion. Moreover, PSMB9 is also related to other immune cell infiltrations, such as macrophages, CD4+ T cells, and B cells, which need to be verified by further experiments. These results show that PSMB9 has a great impact on the tumor immune microenvironment.

### Prediction of the Effect of Immunotherapy in Patients With Glioma

Due to the lack of immune response cohorts for patients with LGG, we use the anti-PD-L1 cohort (IMvigor210) and a combined anti-PD-L1 and anti-CTLA-4 cohort (Gide2019) for further identification of the prognostic value of PSMB9. We divided the cohort into two subgroups according to the expression of PSMB9, and we performed the survival curves that identified that the higher expression of PSMB9 predicts a better prognosis in patients with melanoma (**Figure 7F**). The results of anti-PD-L1 clinical immunotherapy can be divided into 4 groups, which are described previously. According to whether there is a therapeutic effect, we divided the 4 groups into two subgroups, namely, the effective group (PR/CR) and the ineffective group (PD/SD). The immune response of patients’ anti-PD-L1 was better in the PSMB9 high expression group than in the lower expression group. We divided the different treatment responses into two groups and found that the expression of PSMB9 gene was statistically different between the two groups. In the subgroups with effects on the treatment, the expression of PSMB9 was significantly increased (**Figure 7G**). In addition, in the PSMB9 high expression group, the proportions of effective and ineffective treatment were 39.8% and 60.2%, respectively, compared with 16.3% in the PSMB9 low expression group. Additionally, 83.7% form a clear contrast (**Figure 7H**). It shows that PSMB9 has the potential to be used as an immunotherapy target for tumor treatment. The above results show that compared with the low PSMB9 expression group, the anti-PD-L1 treatment effect in the high PSMB9 expression group was more robust, indicating that PSMB9 can assist immunotherapy to a certain extent. It is deduced that PSMB9 protein may participate in the presentation of antigen proteins to reduce the immune escape of tumor cells, so it has great potential for immunotherapy. The survival analysis was performed in the Gide2019 cohort to identify the prognostic role of PSMB9 in immunotherapy, and analogous results were concluded (**Figures 7I, J**). Therefore, based on the response of the two abovementioned treatment cohorts, PSMB9 can be basically concluded as an effective and stable positive prognostic factor for tumor immunotherapy. The proportions of effective and ineffective treatment in the two subgroups also enhanced the conclusion that patients with higher PSMB9 expression receive better curative effects through immunotherapy (**Figure 7K**).

## Discussion

Clinical treatment for glioma is relatively lacking, and with the existence of the blood–brain barrier (BBB), conventional treatment programs are difficult to work in patients with glioma, and conventional surgical treatment has difficulty achieving satisfactory results. The therapeutic effect achieved by various chemotherapy drugs is also very limited (31, 32). Therefore, patients with glioma have a high mortality rate and a very poor prognosis. New tumor prognostic markers and treatment options are in urgent need of development. Therefore, we conducted this study to explore the feasibility of PSMB9 as a clinical prognostic marker for analysis.

In this study, a total of 1,265 samples from three databases were included to confirm the possibility of PSMB9 as a prognostic marker. First, we performed survival analysis and molecular characteristic relevant analysis in three independent cohorts to test the association between PSMB9 expression and the prognosis of patients. We realized that the patients with higher PSMB9 expression have a poorer prognosis. Then we carried out the multivariate Cox regression to ascertain the independent prognostic value of PSMB9. The outcome of AUC curves made further consolidation of PSMB9 as a prognostic factor; calibration curves and DCA curves were conducted to validate the nomogram model, and the results seem to be strongly stable. Next, we focused on the function of PSMB9; thus GO, KEGG, and GSEA were carried out, and evidence showed that PSMB9 participates in a series of immune-related activities. Therefore, ssGSEA, CIBERSORT, ESTIMATE, and immune checkpoint expression analyses were applied to reveal the immune characteristics, which are described in the previous text. At last, we investigated the relationship between PSMB9 expression and the prognosis of patients who received immunotherapy in two immunotherapy cohorts; surprisingly, after immunotherapy, the patients with higher PSMB9 expression have better prognoses, which is opposite to the result in TCGA dataset. This fact shows that PSMB9 functions differently in the different tumors, which matched the previous conclusion, and the consequence is the result of co-regulation by various components of the tumor microenvironment, but its ability as a prognostic marker in tumors is well established.

As one of the important components of proteasome, PSMB9 mainly plays a role in protein degradation. In addition to ubiquitin–proteasome degradation of abnormal or redundant proteins, the proteasome can also participate in a variety of cell biological processes, like cell cycle, apoptosis, and oxidative stress (33). Here we mainly discuss its role in the development of tumors. In the progression of cancer, the proteasome is highly expressed to specifically degrade tumor suppressor factors and block cell apoptosis, thus playing a protective role in tumor cells and promoting tumor progression (34). Therefore, the expression level of PSMB9 was increased in almost tumor tissues, and the higher the level, the more the expression.

Solid tumors consist mainly of tumor cells, with stromal cells and immune cells infiltrating, which constitute the tumor microenvironment (35, 36). The tumor microenvironment is the surrounding environment of tumor cell growth, including not only tumor cells but also surrounding neovascularization, immune cells, and various cell signaling molecules. The complex and diverse biological composition of the tumor microenvironment leads to the complexity of its function, and different tumor cells exist in different tumor microenvironments (37). Researchers have compared the effect of the tumor microenvironment to the relationship between soil and seed (38). Generally speaking, tumor cells are highly amplified, and the blood supply of cells is greatly reduced so that tumor cells can carry out anaerobic glycolysis (39). This effect can act on the immune microenvironment, which can make surrounding cells release a large number of cytokines and chemokines, affecting the tumor microenvironment (40). Chemotactic immune cells acting on tumor cells can affect the growth and development of cancer cells and mediate immune tolerance (41). Many cancer treatment options require a good grasp of the tumor microenvironment. For example, immunotherapy represented by the latest immune checkpoint blockade therapy must be adapted to the tumor microenvironment to function well (42). The study of tumor microenvironment enables us to have a better understanding of tumor cell growth and provides us with research on tumor treatment strategies.

In summary, we constructed a risk model based on the expression level of PSMB9. Based on relevant clinical information, we conducted univariate and multivariate regression analyses to find out if PSMB9 is a possible independent prognostic factor of LGG, and we constructed a nomogram of the prognosis of LGG patients to verify its credibility as a prognostic factor. The results of the enrichment analysis make us focus on its role in tumor immunity. For this reason, we conducted an immune infiltration analysis and proved that the distribution of immune cells in the PSMB9 high and low expression groups is different and the expression of common immune checkpoints is also significantly statistical differences. By verifying the immunotherapy effect of PSMB9 in the melanoma cohort, we proved its promising prospect as a tumor immunotherapy target. Therefore, PSMB9 protein may have great potential for the prognosis of LGG.

## Data Availability Statement

The original contributions presented in the study are included in the article/**Supplementary Material**. Further inquiries can be directed to the corresponding authors.

## Ethics Statement

The studies involving human participants were reviewed and approved by the Medical Ethics Committee of the Second Affiliated Hospital of Nanchang University. The patients/participants provided their written informed consent to participate in this study. Written informed consent was obtained from the individual(s) for the publication of any potentially identifiable images or data included in this article.

## Author Contributions

XZ, KH, and MY designed the study. JZL and LY conducted the data analysis, image stitching, and manuscript writing. JYL and XL collected the clinical samples and revised the manuscript. XY performed the Western blotting and immunofluorescence experiments. QJ conducted the immunofluorescence staining of tumor pathological sections. All authors contributed to the manuscript and approved it for submission.

## Funding

Our research is supported by the National Natural Science Foundation (grant nos. 82002660, 82172989, and 81760445), the Jiangxi Key research and development projects-Key Project (20212BBG71012), Jiangxi Provincial Science and Technology Innovation Base Plan-Provincial Key Laboratory (20212BCD42008), and Province-Youth Talent Project (20212BCJ23023).

## Conflict of Interest

The authors declare that the research was conducted in the absence of any commercial or financial relationships that could be construed as a potential conflict of interest.

## Publisher’s Note

All claims expressed in this article are solely those of the authors and do not necessarily represent those of their affiliated organizations, or those of the publisher, the editors and the reviewers. Any product that may be evaluated in this article, or claim that may be made by its manufacturer, is not guaranteed or endorsed by the publisher.
