# Supplementary figures and images for: Immune Characteristics and Prognosis Analysis of the Proteasome 20S Subunit Beta 9 in Lower-Grade Gliomas
Source: Front Oncol. 2022 Jul 19;12:875131. doi: 10.3389/fonc.2022.875131 (PMC9343852; doi:10.3389/fonc.2022.875131)

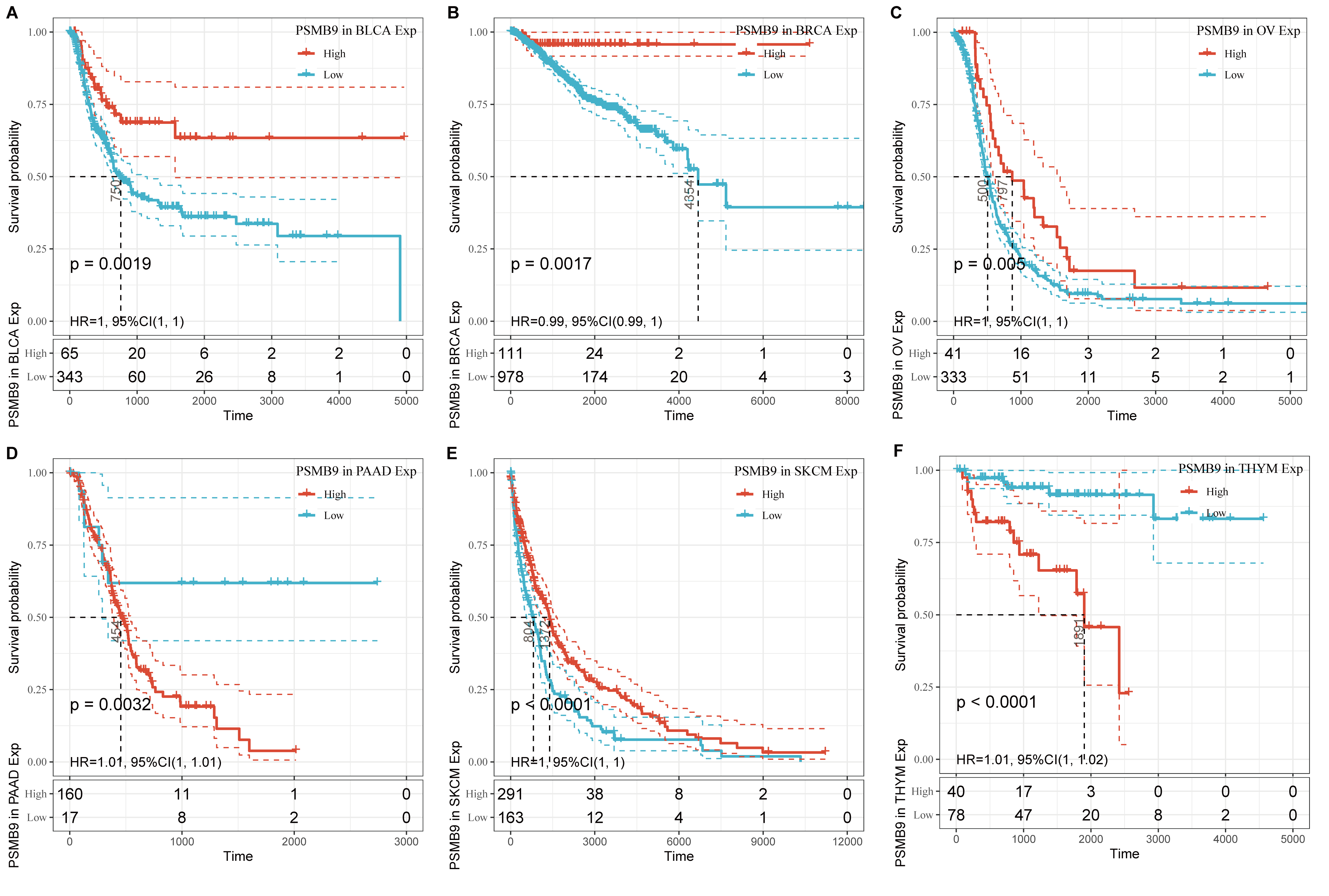

Supplement: Supplementary file 1 [file Image_1.tif]

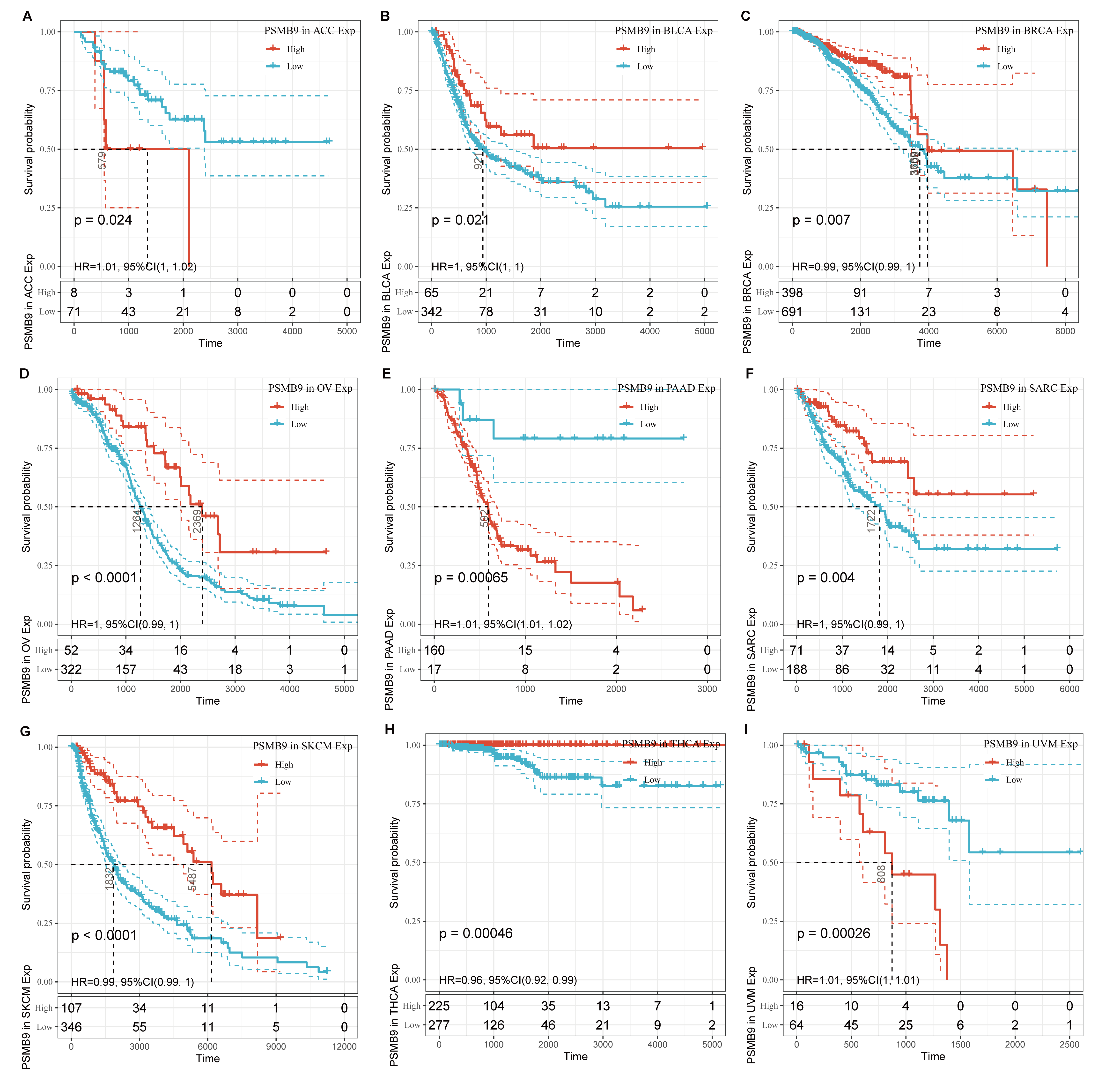

Supplement: Supplementary file 2 [file Image_2.tif]

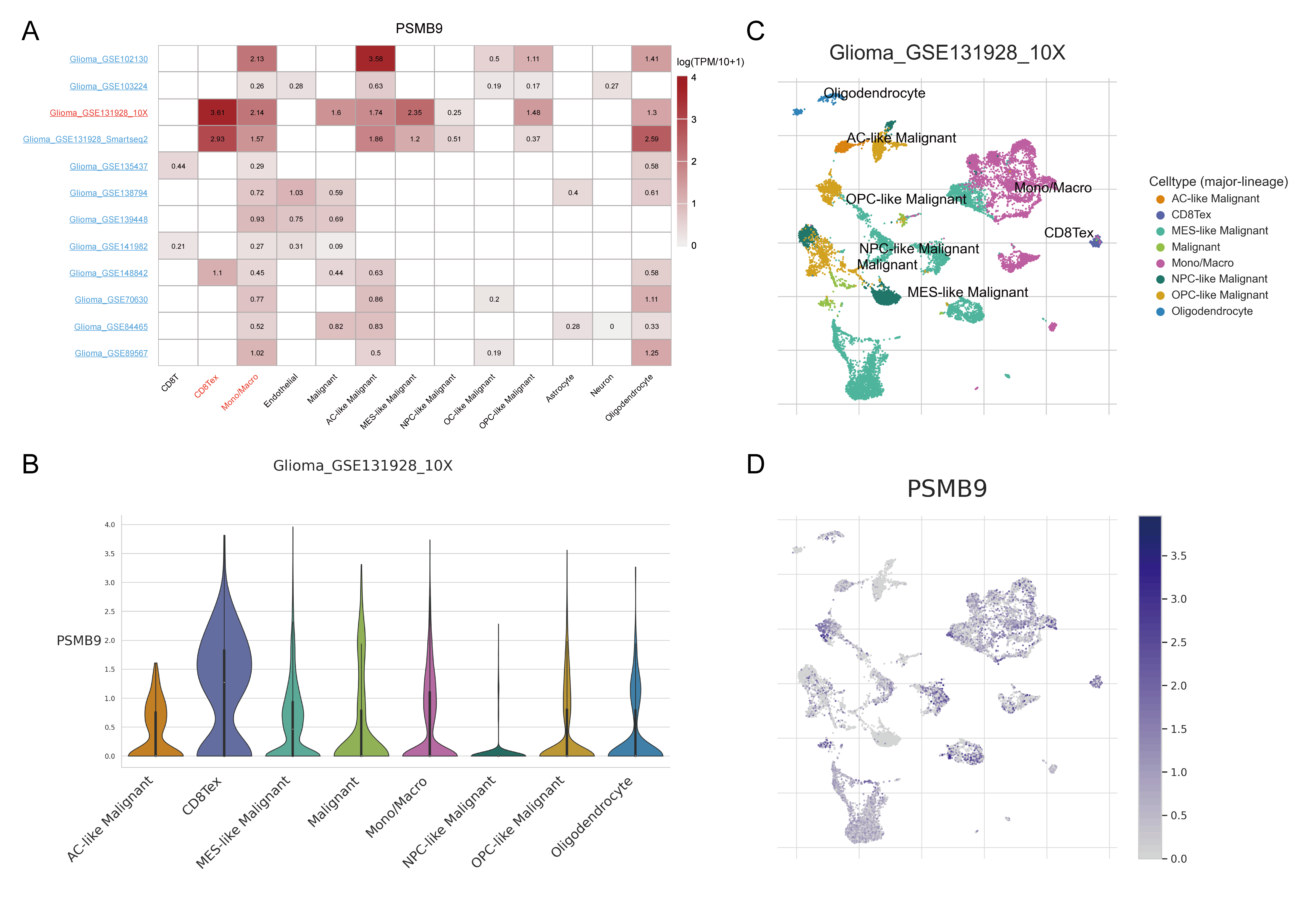

Supplement: Supplementary file 3 [file Image_3.tif]

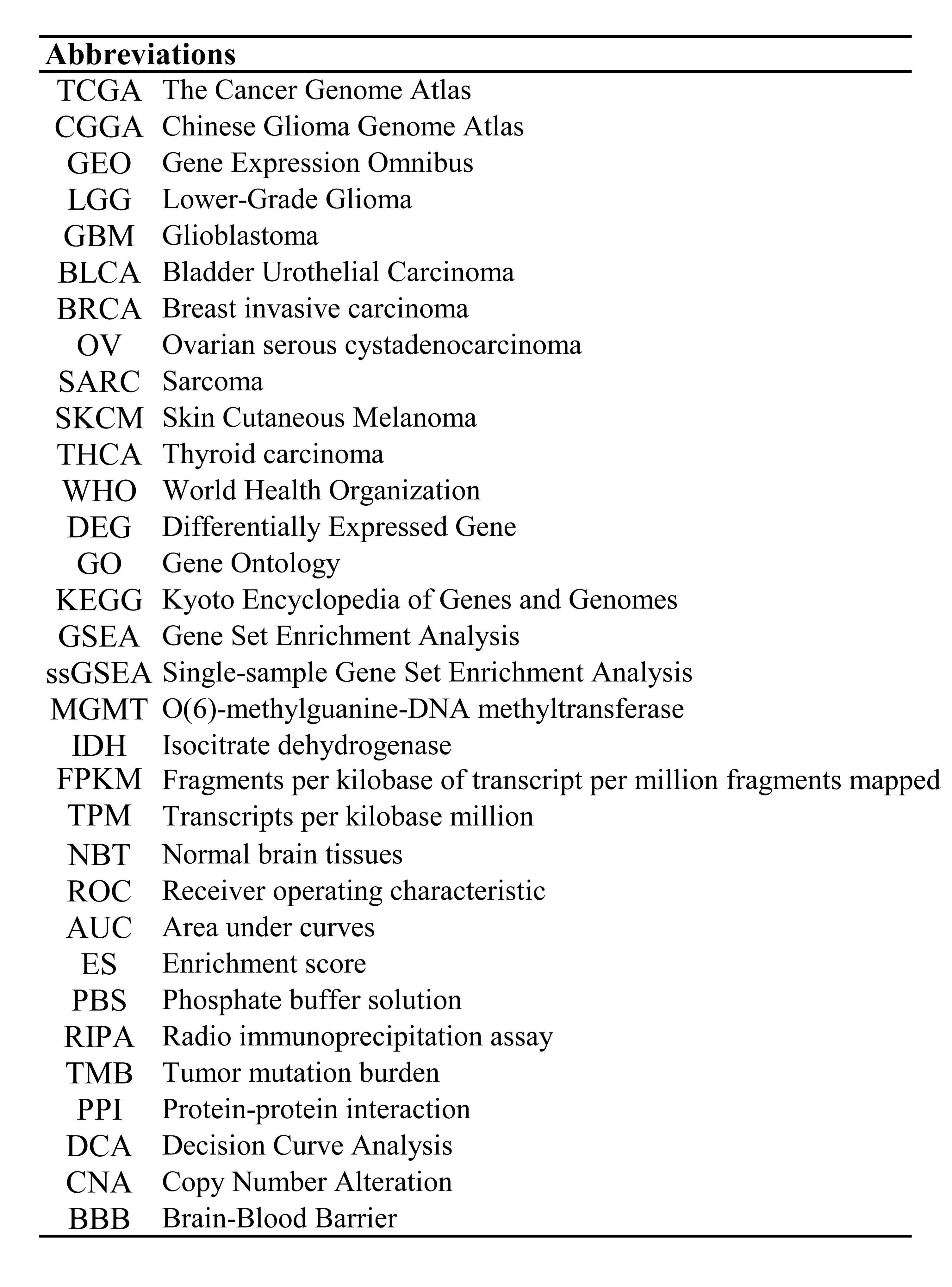

Supplement: Supplementary file 4 [file Image_4.tif]
